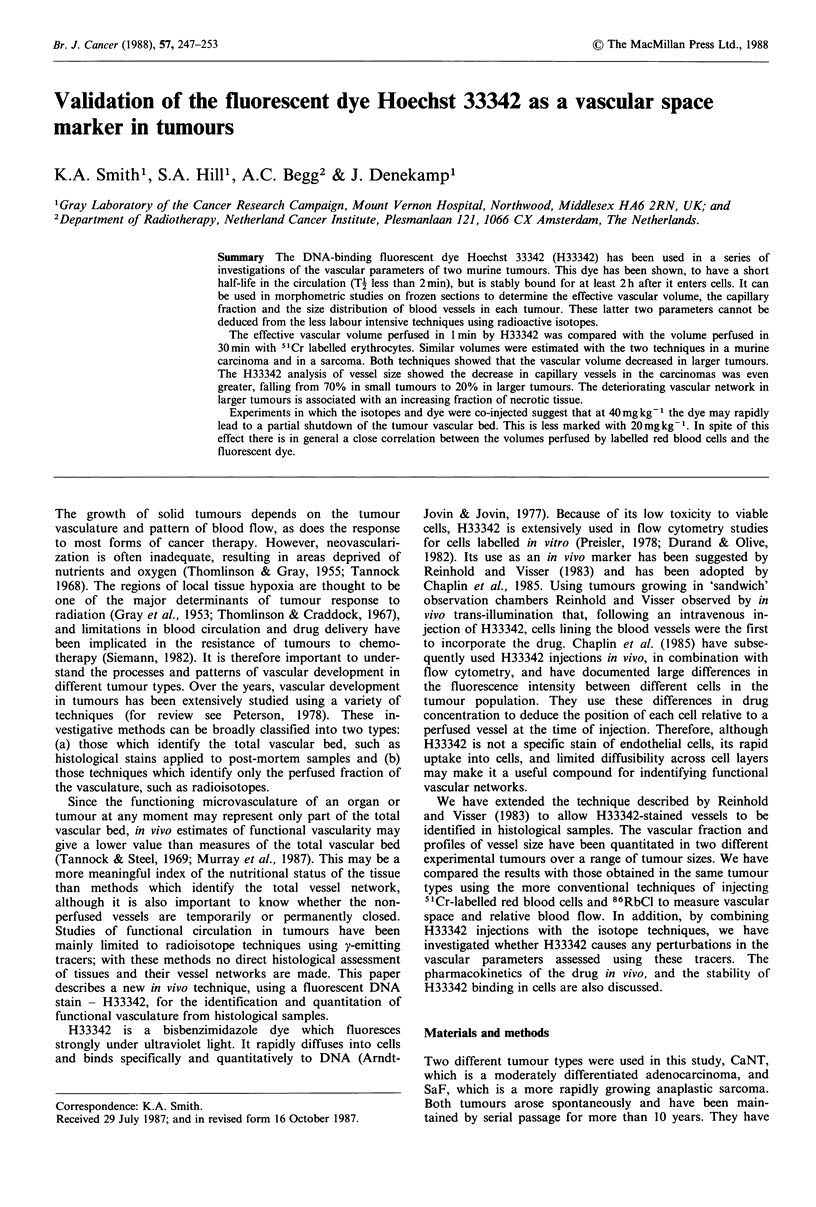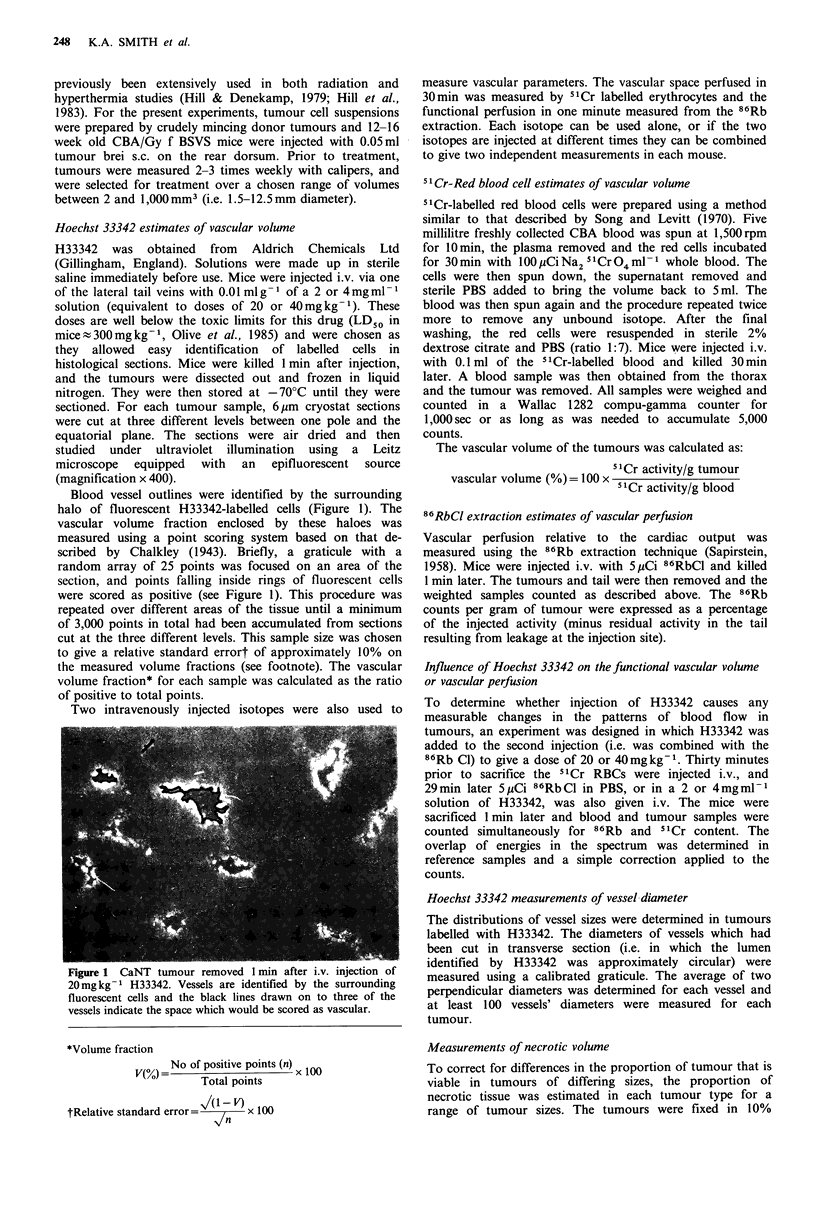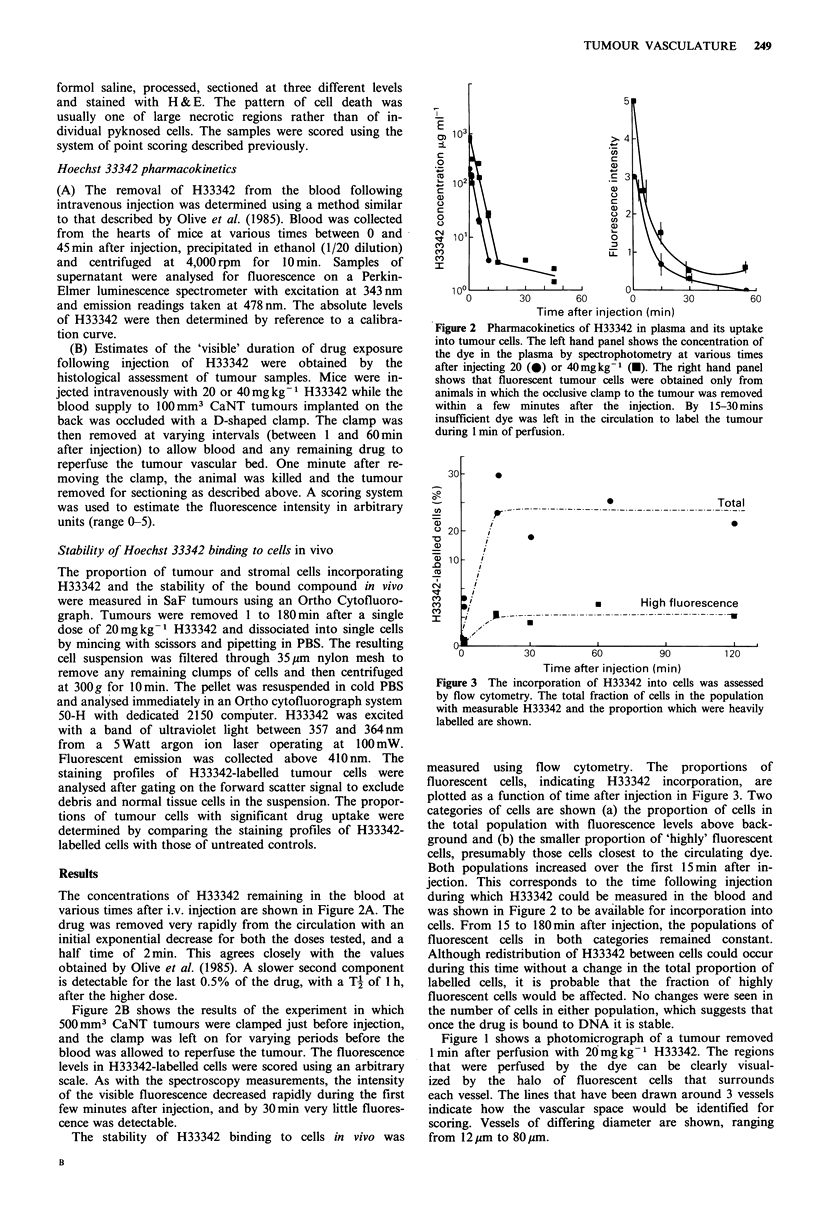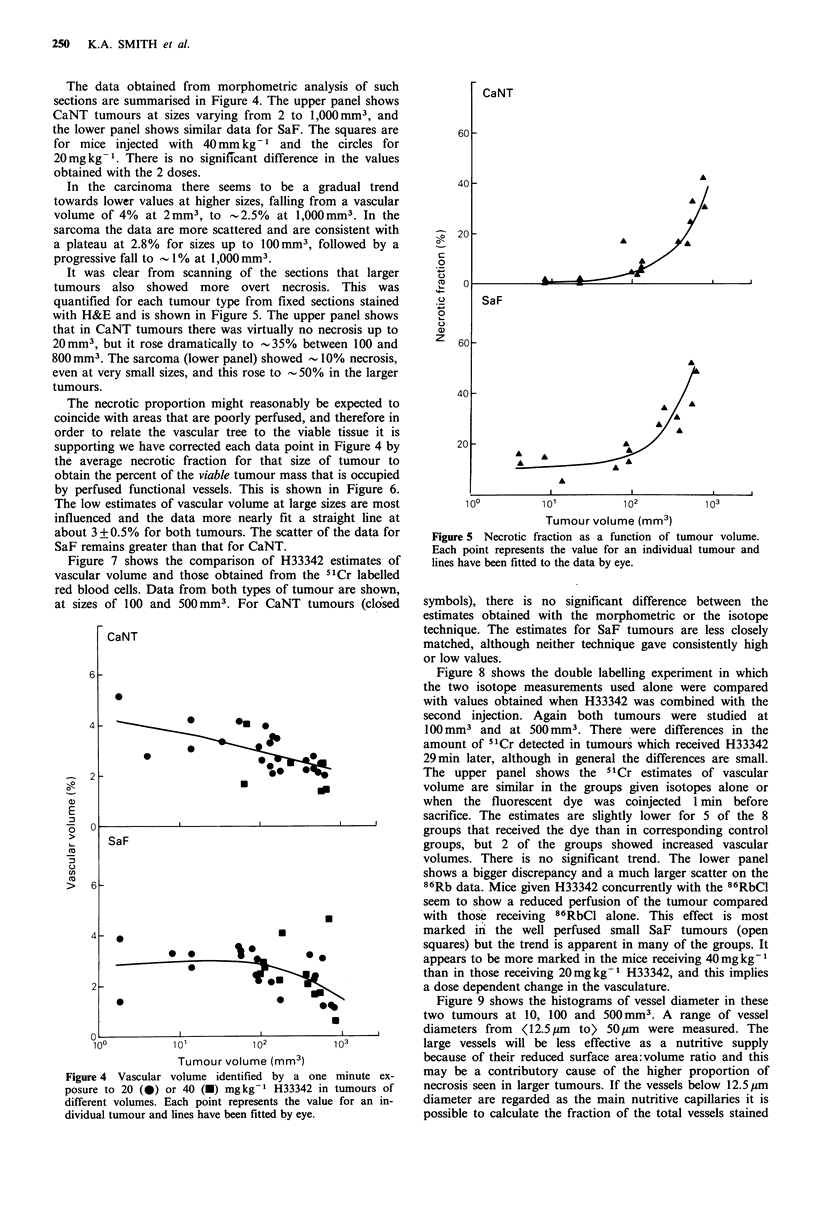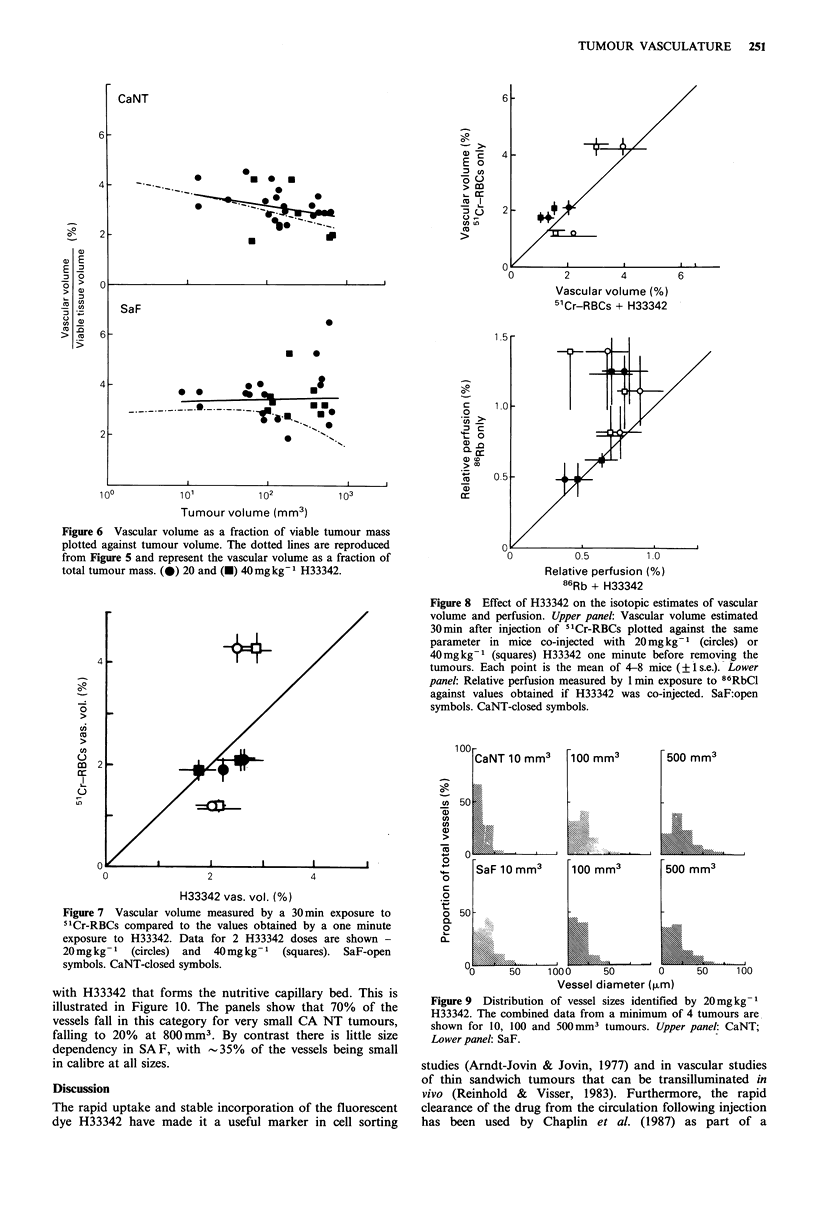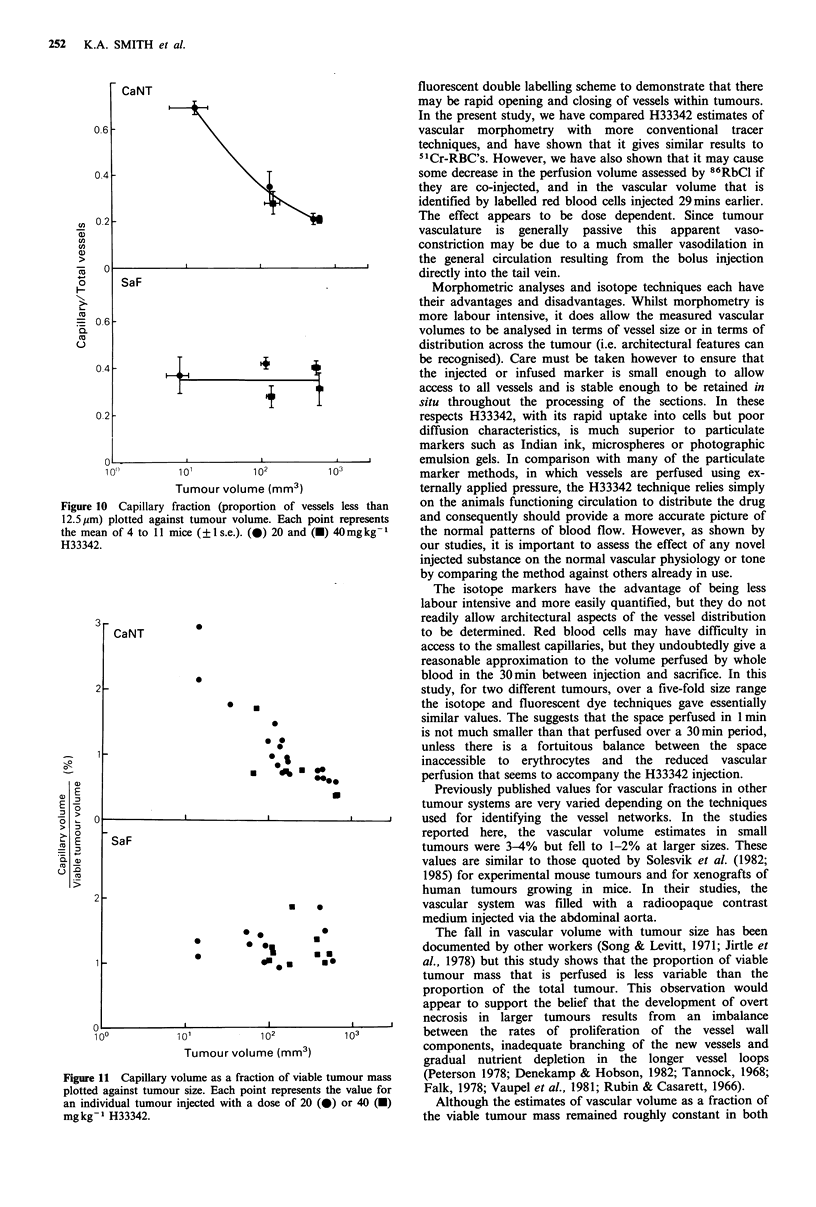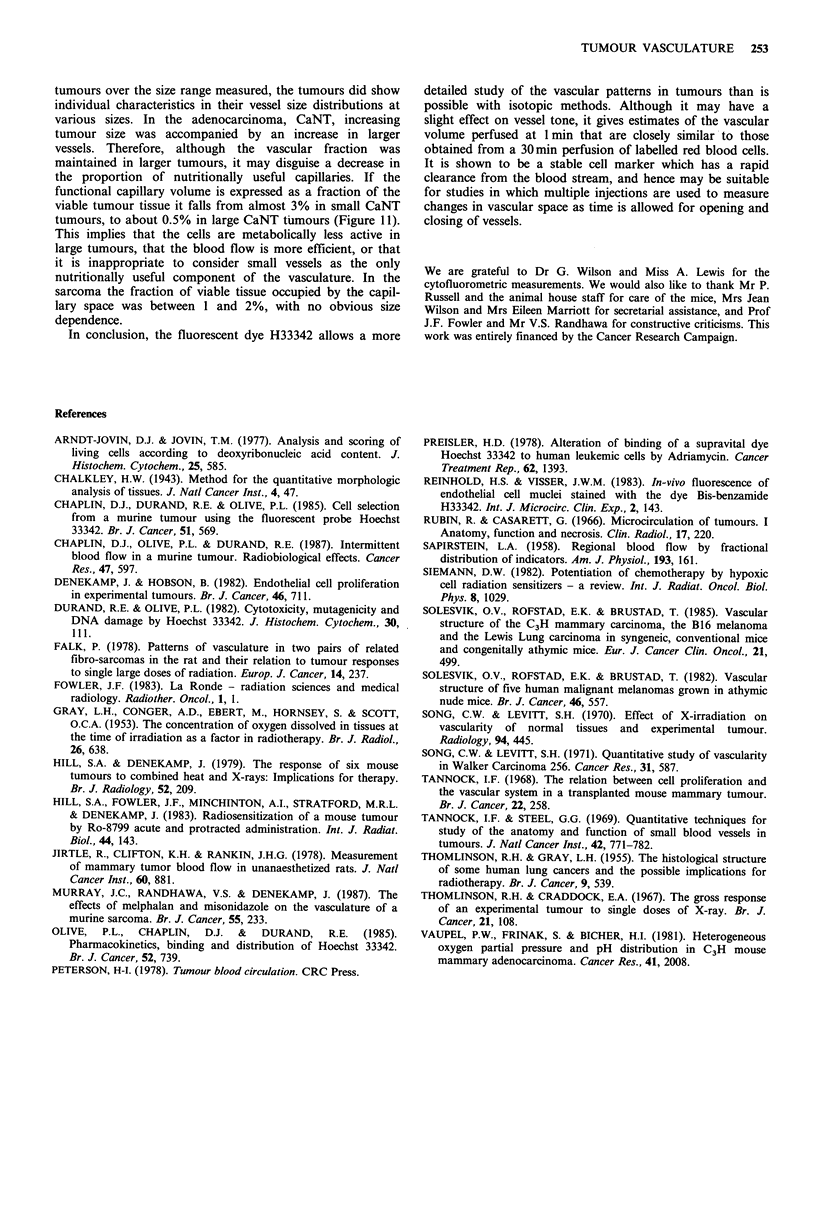# Validation of the fluorescent dye Hoechst 33342 as a vascular space marker in tumours.

**DOI:** 10.1038/bjc.1988.54

**Published:** 1988-03

**Authors:** K. A. Smith, S. A. Hill, A. C. Begg, J. Denekamp

**Affiliations:** Gray Laboratory of the Cancer Research Campaign, Mount Vernon Hospital, Middlesex, UK.

## Abstract

**Images:**


Br. J. Cancer (1988), 57, 247–253 © The MacMillan Press Ltd., 1988

### Validation of the fluorescent dye Hoechst 33342 as a vascular space marker in tumours

K.A. Smith[1], S.A. Hill[1], A.C. Begg[2] & J. Denekamp[1]

[1]*Gray Laboratory of the Cancer Research Campaign, Mount Vernon Hospital, Northwood, Middlesex HA6 2RN, UK; and* [2]*Department of Radiotherapy, Netherland Cancer Institute, Plesmanlaan 121, 1066 CX Amsterdam, The Netherlands.*

**Summary** The DNA-binding fluorescent dye Hoechst 33342 (H33342) has been used in a series of investigations of the vascular parameters of two murine tumours. This dye has been shown, to have a short half-life in the circulation ($T\frac{1}{2}$ less than 2 min), but is stably bound for at least 2 h after it enters cells. It can be used in morphometric studies on frozen sections to determine the effective vascular volume, the capillary fraction and the size distribution of blood vessels in each tumour. These latter two parameters cannot be deduced from the less labour intensive techniques using radioactive isotopes.

The effective vascular volume perfused in 1 min by H33342 was compared with the volume perfused in 30 min with $^{51}$Cr labelled erythrocytes. Similar volumes were estimated with the two techniques in a murine carcinoma and in a sarcoma. Both techniques showed that the vascular volume decreased in larger tumours. The H33342 analysis of vessel size showed the decrease in capillary vessels in the carcinomas was even greater, falling from 70% in small tumours to 20% in larger tumours. The deteriorating vascular network in larger tumours is associated with an increasing fraction of necrotic tissue.

Experiments in which the isotopes and dye were co-injected suggest that at 40 mg kg$^{-1}$ the dye may rapidly lead to a partial shutdown of the tumour vascular bed. This is less marked with 20 mg kg$^{-1}$. In spite of this effect there is in general a close correlation between the volumes perfused by labelled red blood cells and the fluorescent dye.

The growth of solid tumours depends on the tumour vasculature and pattern of blood flow, as does the response to most forms of cancer therapy. However, neovascularization is often inadequate, resulting in areas deprived of nutrients and oxygen (Thomlinson & Gray, 1955; Tannock 1968). The regions of local tissue hypoxia are thought to be one of the major determinants of tumour response to radiation (Gray *et al.*, 1953; Thomlinson & Craddock, 1967), and limitations in blood circulation and drug delivery have been implicated in the resistance of tumours to chemotherapy (Siemann, 1982). It is therefore important to understand the processes and patterns of vascular development in different tumour types. Over the years, vascular development in tumours has been extensively studied using a variety of techniques (for review see Peterson, 1978). These investigative methods can be broadly classified into two types: (a) those which identify the total vascular bed, such as histological stains applied to post-mortem samples and (b) those techniques which identify only the perfused fraction of the vasculature, such as radioisotopes.

Since the functioning microvasculature of an organ or tumour at any moment may represent only part of the total vascular bed, *in vivo* estimates of functional vascularity may give a lower value than measures of the total vascular bed (Tannock & Steel, 1969; Murray *et al.*, 1987). This may be a more meaningful index of the nutritional status of the tissue than methods which identify the total vessel network, although it is also important to know whether the non-perfused vessels are temporarily or permanently closed. Studies of functional circulation in tumours have been mainly limited to radioisotope techniques using $\gamma$-emitting tracers; with these methods no direct histological assessment of tissues and their vessel networks are made. This paper describes a new *in vivo* technique, using a fluorescent DNA stain – H33342, for the identification and quantitation of functional vasculature from histological samples.

H33342 is a bisbenzimidazole dye which fluoresces strongly under ultraviolet light. It rapidly diffuses into cells and binds specifically and quantitatively to DNA (Arndt-Jovin & Jovin, 1977). Because of its low toxicity to viable cells, H33342 is extensively used in flow cytometry studies for cells labelled *in vitro* (Preisler, 1978; Durand & Olive, 1982). Its use as an *in vivo* marker has been suggested by Reinhold and Visser (1983) and has been adopted by Chaplin *et al.*, 1985. Using tumours growing in 'sandwich' observation chambers Reinhold and Visser observed by *in vivo* trans-illumination that, following an intravenous injection of H33342, cells lining the blood vessels were the first to incorporate the drug. Chaplin *et al.* (1985) have subsequently used H33342 injections *in vivo*, in combination with flow cytometry, and have documented large differences in the fluorescence intensity between different cells in the tumour population. They use these differences in drug concentration to deduce the position of each cell relative to a perfused vessel at the time of injection. Therefore, although H33342 is not a specific stain of endothelial cells, its rapid uptake into cells, and limited diffusibility across cell layers may make it a useful compound for indentifying functional vascular networks.

We have extended the technique described by Reinhold and Visser (1983) to allow H33342-stained vessels to be identified in histological samples. The vascular fraction and profiles of vessel size have been quantitated in two different experimental tumours over a range of tumour sizes. We have compared the results with those obtained in the same tumour types using the more conventional techniques of injecting $^{51}$Cr-labelled red blood cells and $^{86}$RbCl to measure vascular space and relative blood flow. In addition, by combining H33342 injections with the isotope techniques, we have investigated whether H33342 causes any perturbations in the vascular parameters assessed using these tracers. The pharmacokinetics of the drug *in vivo*, and the stability of H33342 binding in cells are also discussed.

### Materials and methods

Two different tumour types were used in this study, CaNT, which is a moderately differentiated adenocarcinoma, and SaF, which is a more rapidly growing anaplastic sarcoma. Both tumours arose spontaneously and have been maintained by serial passage for more than 10 years. They have

Correspondence: K.A. Smith.
Received 29 July 1987; and in revised form 16 October 1987.

previously been extensively used in both radiation and hyperthermia studies (Hill & Denekamp, 1979; Hill *et al.*, 1983). For the present experiments, tumour cell suspensions were prepared by crudely mincing donor tumours and 12–16 week old CBA/Gy f BSVS mice were injected with 0.05 ml tumour brei s.c. on the rear dorsum. Prior to treatment, tumours were measured 2–3 times weekly with calipers, and were selected for treatment over a chosen range of volumes between 2 and 1,000 $mm^3$ (i.e. 1.5–12.5 mm diameter).

*Hoechst 33342 estimates of vascular volume*

H33342 was obtained from Aldrich Chemicals Ltd (Gillingham, England). Solutions were made up in sterile saline immediately before use. Mice were injected i.v. via one of the lateral tail veins with 0.01 ml $g^{-1}$ of a 2 or 4 mg $ml^{-1}$ solution (equivalent to doses of 20 or 40 mg $kg^{-1}$). These doses are well below the toxic limits for this drug ($LD_{50}$ in mice ≈ 300 mg $kg^{-1}$, Olive *et al.*, 1985) and were chosen as they allowed easy identification of labelled cells in histological sections. Mice were killed 1 min after injection, and the tumours were dissected out and frozen in liquid nitrogen. They were then stored at −70°C until they were sectioned. For each tumour sample, 6 μm cryostat sections were cut at three different levels between one pole and the equatorial plane. The sections were air dried and then studied under ultraviolet illumination using a Leitz microscope equipped with an epifluorescent source (magnification × 400).

Blood vessel outlines were identified by the surrounding halo of fluorescent H33342-labelled cells (Figure 1). The vascular volume fraction enclosed by these haloes was measured using a point scoring system based on that described by Chalkley (1943). Briefly, a graticule with a random array of 25 points was focused on an area of the section, and points falling inside rings of fluorescent cells were scored as positive (see Figure 1). This procedure was repeated over different areas of the tissue until a minimum of 3,000 points in total had been accumulated from sections cut at the three different levels. This sample size was chosen to give a relative standard error† of approximately 10% on the measured volume fractions (see footnote). The vascular volume fraction* for each sample was calculated as the ratio of positive to total points.

Two intravenously injected isotopes were also used to measure vascular parameters. The vascular space perfused in 30 min was measured by $^{51}Cr$ labelled erythrocytes and the functional perfusion in one minute measured from the $^{86}Rb$ extraction. Each isotope can be used alone, or if the two isotopes are injected at different times they can be combined to give two independent measurements in each mouse.

**Figure 1** CaNT tumour removed 1 min after i.v. injection of 20 mg $kg^{-1}$ H33342. Vessels are identified by the surrounding fluorescent cells and the black lines drawn on to three of the vessels indicate the space which would be scored as vascular.

*$^{51}Cr$-Red blood cell estimates of vascular volume*

$^{51}Cr$-labelled red blood cells were prepared using a method similar to that described by Song and Levitt (1970). Five millilitre freshly collected CBA blood was spun at 1,500 rpm for 10 min, the plasma removed and the red cells incubated for 30 min with 100 μCi $Na_2\,^{51}CrO_4$ $ml^{-1}$ whole blood. The cells were then spun down, the supernatant removed and sterile PBS added to bring the volume back to 5 ml. The blood was then spun again and the procedure repeated twice more to remove any unbound isotope. After the final washing, the red cells were resuspended in sterile 2% dextrose citrate and PBS (ratio 1:7). Mice were injected i.v. with 0.1 ml of the $^{51}Cr$-labelled blood and killed 30 min later. A blood sample was then obtained from the thorax and the tumour was removed. All samples were weighed and counted in a Wallac 1282 compu-gamma counter for 1,000 sec or as long as was needed to accumulate 5,000 counts.

The vascular volume of the tumours was calculated as:

$$\text{vascular volume } (\%) = 100 \times \frac{^{51}\text{Cr activity/g tumour}}{^{51}\text{Cr activity/g blood}}$$

*$^{86}RbCl$ extraction estimates of vascular perfusion*

Vascular perfusion relative to the cardiac output was measured using the $^{86}Rb$ extraction technique (Sapirstein, 1958). Mice were injected i.v. with 5 μCi $^{86}RbCl$ and killed 1 min later. The tumours and tail were then removed and the weighted samples counted as described above. The $^{86}Rb$ counts per gram of tumour were expressed as a percentage of the injected activity (minus residual activity in the tail resulting from leakage at the injection site).

*Influence of Hoechst 33342 on the functional vascular volume or vascular perfusion*

To determine whether injection of H33342 causes any measurable changes in the patterns of blood flow in tumours, an experiment was designed in which H33342 was added to the second injection (i.e. was combined with the $^{86}Rb$ Cl) to give a dose of 20 or 40 mg $kg^{-1}$. Thirty minutes prior to sacrifice the $^{51}Cr$ RBCs were injected i.v., and 29 min later 5 μCi $^{86}RbCl$ in PBS, or in a 2 or 4 mg $ml^{-1}$ solution of H33342, was also given i.v. The mice were sacrificed 1 min later and blood and tumour samples were counted simultaneously for $^{86}Rb$ and $^{51}Cr$ content. The overlap of energies in the spectrum was determined in reference samples and a simple correction applied to the counts.

*Hoechst 33342 measurements of vessel diameter*

The distributions of vessel sizes were determined in tumours labelled with H33342. The diameters of vessels which had been cut in transverse section (i.e. in which the lumen identified by H33342 was approximately circular) were measured using a calibrated graticule. The average of two perpendicular diameters was determined for each vessel and at least 100 vessels' diameters were measured for each tumour.

*Measurements of necrotic volume*

To correct for differences in the proportion of tumour that is viable in tumours of differing sizes, the proportion of necrotic tissue was estimated in each tumour type for a range of tumour sizes. The tumours were fixed in 10%

*Volume fraction

$$V(\%) = \frac{\text{No of positive points } (n)}{\text{Total points}} \times 100$$

†Relative standard error $= \frac{\sqrt{(1-V)}}{\sqrt{n}} \times 100$

formol saline, processed, sectioned at three different levels and stained with H&E. The pattern of cell death was usually one of large necrotic regions rather than of individual pyknosed cells. The samples were scored using the system of point scoring described previously.

*Hoechst 33342 pharmacokinetics*

(A) The removal of H33342 from the blood following intravenous injection was determined using a method similar to that described by Olive *et al.* (1985). Blood was collected from the hearts of mice at various times between 0 and 45 min after injection, precipitated in ethanol (1/20 dilution) and centrifuged at 4,000 rpm for 10 min. Samples of supernatant were analysed for fluorescence on a Perkin-Elmer luminescence spectrometer with excitation at 343 nm and emission readings taken at 478 nm. The absolute levels of H33342 were then determined by reference to a calibration curve.

(B) Estimates of the 'visible' duration of drug exposure following injection of H33342 were obtained by the histological assessment of tumour samples. Mice were injected intravenously with 20 or 40 mg kg$^{-1}$ H33342 while the blood supply to 100 mm$^3$ CaNT tumours implanted on the back was occluded with a D-shaped clamp. The clamp was then removed at varying intervals (between 1 and 60 min after injection) to allow blood and any remaining drug to reperfuse the tumour vascular bed. One minute after removing the clamp, the animal was killed and the tumour removed for sectioning as described above. A scoring system was used to estimate the fluorescence intensity in arbitrary units (range 0–5).

*Stability of Hoechst 33342 binding to cells* in vivo

The proportion of tumour and stromal cells incorporating H33342 and the stability of the bound compound *in vivo* were measured in SaF tumours using an Ortho Cytofluorograph. Tumours were removed 1 to 180 min after a single dose of 20 mg kg$^{-1}$ H33342 and dissociated into single cells by mincing with scissors and pipetting in PBS. The resulting cell suspension was filtered through 35 $\mu$m nylon mesh to remove any remaining clumps of cells and then centrifuged at 300 *g* for 10 min. The pellet was resuspended in cold PBS and analysed immediately in an Ortho cytofluorograph system 50-H with dedicated 2150 computer. H33342 was excited with a band of ultraviolet light between 357 and 364 nm from a 5 Watt argon ion laser operating at 100 mW. Fluorescent emission was collected above 410 nm. The staining profiles of H33342-labelled tumour cells were analysed after gating on the forward scatter signal to exclude debris and normal tissue cells in the suspension. The proportions of tumour cells with significant drug uptake were determined by comparing the staining profiles of H33342-labelled cells with those of untreated controls.

### Results

The concentrations of H33342 remaining in the blood at various times after i.v. injection are shown in Figure 2A. The drug was removed very rapidly from the circulation with an initial exponential decrease for both the doses tested, and a half time of 2 min. This agrees closely with the values obtained by Olive *et al.* (1985). A slower second component is detectable for the last 0.5% of the drug, with a T$\frac{1}{2}$ of 1 h, after the higher dose.

Figure 2B shows the results of the experiment in which 500 mm$^3$ CaNT tumours were clamped just before injection, and the clamp was left on for varying periods before the blood was allowed to reperfuse the tumour. The fluorescence levels in H33342-labelled cells were scored using an arbitrary scale. As with the spectroscopy measurements, the intensity of the visible fluorescence decreased rapidly during the first few minutes after injection, and by 30 min very little fluorescence was detectable.

The stability of H33342 binding to cells *in vivo* was measured using flow cytometry. The proportions of fluorescent cells, indicating H33342 incorporation, are plotted as a function of time after injection in Figure 3. Two categories of cells are shown (a) the proportion of cells in the total population with fluorescence levels above background and (b) the smaller proportion of 'highly' fluorescent cells, presumably those cells closest to the circulating dye. Both populations increased over the first 15 min after injection. This corresponds to the time following injection during which H33342 could be measured in the blood and was shown in Figure 2 to be available for incorporation into cells. From 15 to 180 min after injection, the populations of fluorescent cells in both categories remained constant. Although redistribution of H33342 between cells could occur during this time without a change in the total proportion of labelled cells, it is probable that the fraction of highly fluorescent cells would be affected. No changes were seen in the number of cells in either population, which suggests that once the drug is bound to DNA it is stable.

Figure 1 shows a photomicrograph of a tumour removed 1 min after perfusion with 20 mg kg$^{-1}$ H33342. The regions that were perfused by the dye can be clearly visualized by the halo of fluorescent cells that surrounds each vessel. The lines that have been drawn around 3 vessels indicate how the vascular space would be identified for scoring. Vessels of differing diameter are shown, ranging from 12 $\mu$m to 80 $\mu$m.

**Figure 2** Pharmacokinetics of H33342 in plasma and its uptake into tumour cells. The left hand panel shows the concentration of the dye in the plasma by spectrophotometry at various times after injecting 20 (●) or 40 mg kg$^{-1}$ (■). The right hand panel shows that fluorescent tumour cells were obtained only from animals in which the occlusive clamp to the tumour was removed within a few minutes after the injection. By 15–30 mins insufficient dye was left in the circulation to label the tumour during 1 min of perfusion.

**Figure 3** The incorporation of H33342 into cells was assessed by flow cytometry. The total fraction of cells in the population with measurable H33342 and the proportion which were heavily labelled are shown.

The data obtained from morphometric analysis of such sections are summarised in Figure 4. The upper panel shows CaNT tumours at sizes varying from 2 to 1,000 $mm^3$, and the lower panel shows similar data for SaF. The squares are for mice injected with 40 mm $kg^{-1}$ and the circles for 20 mg $kg^{-1}$. There is no significant difference in the values obtained with the 2 doses.

In the carcinoma there seems to be a gradual trend towards lower values at higher sizes, falling from a vascular volume of 4% at 2 $mm^3$, to ~2.5% at 1,000 $mm^3$. In the sarcoma the data are more scattered and are consistent with a plateau at 2.8% for sizes up to 100 $mm^3$, followed by a progressive fall to ~1% at 1,000 $mm^3$.

It was clear from scanning of the sections that larger tumours also showed more overt necrosis. This was quantified for each tumour type from fixed sections stained with H&E and is shown in Figure 5. The upper panel shows that in CaNT tumours there was virtually no necrosis up to 20 $mm^3$, but it rose dramatically to ~35% between 100 and 800 $mm^3$. The sarcoma (lower panel) showed ~10% necrosis, even at very small sizes, and this rose to ~50% in the larger tumours.

The necrotic proportion might reasonably be expected to coincide with areas that are poorly perfused, and therefore in order to relate the vascular tree to the viable tissue it is supporting we have corrected each data point in Figure 4 by the average necrotic fraction for that size of tumour to obtain the percent of the *viable* tumour mass that is occupied by perfused functional vessels. This is shown in Figure 6. The low estimates of vascular volume at large sizes are most influenced and the data more nearly fit a straight line at about $3 \pm 0.5\%$ for both tumours. The scatter of the data for SaF remains greater than that for CaNT.

Figure 7 shows the comparison of H33342 estimates of vascular volume and those obtained from the $^{51}Cr$ labelled red blood cells. Data from both types of tumour are shown, at sizes of 100 and 500 $mm^3$. For CaNT tumours (closed symbols), there is no significant difference between the estimates obtained with the morphometric or the isotope technique. The estimates for SaF tumours are less closely matched, although neither technique gave consistently high or low values.

**Figure 4** Vascular volume identified by a one minute exposure to 20 (●) or 40 (■) mg $kg^{-1}$ H33342 in tumours of different volumes. Each point represents the value for an individual tumour and lines have been fitted by eye.

**Figure 5** Necrotic fraction as a function of tumour volume. Each point represents the value for an individual tumour and lines have been fitted to the data by eye.

Figure 8 shows the double labelling experiment in which the two isotope measurements used alone were compared with values obtained when H33342 was combined with the second injection. Again both tumours were studied at 100 $mm^3$ and at 500 $mm^3$. There were differences in the amount of $^{51}Cr$ detected in tumours which received H33342 29 min later, although in general the differences are small. The upper panel shows the $^{51}Cr$ estimates of vascular volume are similar in the groups given isotopes alone or when the fluorescent dye was coinjected 1 min before sacrifice. The estimates are slightly lower for 5 of the 8 groups that received the dye than in corresponding control groups, but 2 of the groups showed increased vascular volumes. There is no significant trend. The lower panel shows a bigger discrepancy and a much larger scatter on the $^{86}Rb$ data. Mice given H33342 concurrently with the $^{86}RbCl$ seem to show a reduced perfusion of the tumour compared with those receiving $^{86}RbCl$ alone. This effect is most marked in the well perfused small SaF tumours (open squares) but the trend is apparent in many of the groups. It appears to be more marked in the mice receiving 40 mg $kg^{-1}$ than in those receiving 20 mg $kg^{-1}$ H33342, and this implies a dose dependent change in the vasculature.

Figure 9 shows the histograms of vessel diameter in these two tumours at 10, 100 and 500 $mm^3$. A range of vessel diameters from (<12.5 μm to> 50 μm were measured. The large vessels will be less effective as a nutritive supply because of their reduced surface area:volume ratio and this may be a contributory cause of the higher proportion of necrosis seen in larger tumours. If the vessels below 12.5 μm diameter are regarded as the main nutritive capillaries it is possible to calculate the fraction of the total vessels stained

**Figure 6** Vascular volume as a fraction of viable tumour mass plotted against tumour volume. The dotted lines are reproduced from **Figure 5** and represent the vascular volume as a fraction of total tumour mass. (●) 20 and (■) 40 mg kg$^{-1}$ H33342.

**Figure 7** Vascular volume measured by a 30 min exposure to $^{51}$Cr-RBCs compared to the values obtained by a one minute exposure to H33342. Data for 2 H33342 doses are shown – 20 mg kg$^{-1}$ (circles) and 40 mg kg$^{-1}$ (squares). SaF-open symbols. CaNT-closed symbols.

with H33342 that forms the nutritive capillary bed. This is illustrated in Figure 10. The panels show that 70% of the vessels fall in this category for very small CA NT tumours, falling to 20% at 800 mm$^3$. By contrast there is little size dependency in SA F, with ~35% of the vessels being small in calibre at all sizes.

### Discussion

The rapid uptake and stable incorporation of the fluorescent dye H33342 have made it a useful marker in cell sorting

**Figure 8** Effect of H33342 on the isotopic estimates of vascular volume and perfusion. *Upper panel*: Vascular volume estimated 30 min after injection of $^{51}$Cr-RBCs plotted against the same parameter in mice co-injected with 20 mg kg$^{-1}$ (circles) or 40 mg kg$^{-1}$ (squares) H33342 one minute before removing the tumours. Each point is the mean of 4–8 mice (±1 s.e.). *Lower panel*: Relative perfusion measured by 1 min exposure to $^{86}$RbCl against values obtained if H33342 was co-injected. SaF:open symbols. CaNT-closed symbols.

**Figure 9** Distribution of vessel sizes identified by 20 mg kg$^{-1}$ H33342. The combined data from a minimum of 4 tumours are shown for 10, 100 and 500 mm$^3$ tumours. *Upper panel*: CaNT; *Lower panel*: SaF.

studies (Arndt-Jovin & Jovin, 1977) and in vascular studies of thin sandwich tumours that can be transilluminated *in vivo* (Reinhold & Visser, 1983). Furthermore, the rapid clearance of the drug from the circulation following injection has been used by Chaplin *et al.* (1987) as part of a

**Figure 10** Capillary fraction (proportion of vessels less than 12.5 μm) plotted against tumour volume. Each point represents the mean of 4 to 11 mice (±1 s.e.). (●) 20 and (■) 40 mg kg$^{-1}$ H33342.

**Figure 11** Capillary volume as a fraction of viable tumour mass plotted against tumour size. Each point represents the value for an individual tumour injected with a dose of 20 (●) or 40 (■) mg kg$^{-1}$ H33342.

fluorescent double labelling scheme to demonstrate that there may be rapid opening and closing of vessels within tumours. In the present study, we have compared H33342 estimates of vascular morphometry with more conventional tracer techniques, and have shown that it gives similar results to $^{51}$Cr-RBC's. However, we have also shown that it may cause some decrease in the perfusion volume assessed by $^{86}$RbCl if they are co-injected, and in the vascular volume that is identified by labelled red blood cells injected 29 mins earlier. The effect appears to be dose dependent. Since tumour vasculature is generally passive this apparent vasoconstriction may be due to a much smaller vasodilation in the general circulation resulting from the bolus injection directly into the tail vein.

Morphometric analyses and isotope techniques each have their advantages and disadvantages. Whilst morphometry is more labour intensive, it does allow the measured vascular volumes to be analysed in terms of vessel size or in terms of distribution across the tumour (i.e. architectural features can be recognised). Care must be taken however to ensure that the injected or infused marker is small enough to allow access to all vessels and is stable enough to be retained *in situ* throughout the processing of the sections. In these respects H33342, with its rapid uptake into cells but poor diffusion characteristics, is much superior to particulate markers such as Indian ink, microspheres or photographic emulsion gels. In comparison with many of the particulate marker methods, in which vessels are perfused using externally applied pressure, the H33342 technique relies simply on the animals functioning circulation to distribute the drug and consequently should provide a more accurate picture of the normal patterns of blood flow. However, as shown by our studies, it is important to assess the effect of any novel injected substance on the normal vascular physiology or tone by comparing the method against others already in use.

The isotope markers have the advantage of being less labour intensive and more easily quantified, but they do not readily allow architectural aspects of the vessel distribution to be determined. Red blood cells may have difficulty in access to the smallest capillaries, but they undoubtedly give a reasonable approximation to the volume perfused by whole blood in the 30 min between injection and sacrifice. In this study, for two different tumours, over a five-fold size range the isotope and fluorescent dye techniques gave essentially similar values. The suggests that the space perfused in 1 min is not much smaller than that perfused over a 30 min period, unless there is a fortuitous balance between the space inaccessible to erythrocytes and the reduced vascular perfusion that seems to accompany the H33342 injection.

Previously published values for vascular fractions in other tumour systems are very varied depending on the techniques used for identifying the vessel networks. In the studies reported here, the vascular volume estimates in small tumours were 3–4% but fell to 1–2% at larger sizes. These values are similar to those quoted by Solesvik *et al.* (1982; 1985) for experimental mouse tumours and for xenografts of human tumours growing in mice. In their studies, the vascular system was filled with a radioopaque contrast medium injected via the abdominal aorta.

The fall in vascular volume with tumour size has been documented by other workers (Song & Levitt, 1971; Jirtle *et al.*, 1978) but this study shows that the proportion of viable tumour mass that is perfused is less variable than the proportion of the total tumour. This observation would appear to support the belief that the development of overt necrosis in larger tumours results from an imbalance between the rates of proliferation of the vessel wall components, inadequate branching of the new vessels and gradual nutrient depletion in the longer vessel loops (Peterson 1978; Denekamp & Hobson, 1982; Tannock, 1968; Falk, 1978; Vaupel *et al.*, 1981; Rubin & Casarett, 1966).

Although the estimates of vascular volume as a fraction of the viable tumour mass remained roughly constant in both tumours over the size range measured, the tumours did show individual characteristics in their vessel size distributions at various sizes. In the adenocarcinoma, CaNT, increasing tumour size was accompanied by an increase in larger vessels. Therefore, although the vascular fraction was maintained in larger tumours, it may disguise a decrease in the proportion of nutritionally useful capillaries. If the functional capillary volume is expressed as a fraction of the viable tumour tissue it falls from almost 3% in small CaNT tumours, to about 0.5% in large CaNT tumours (Figure 11). This implies that the cells are metabolically less active in large tumours, that the blood flow is more efficient, or that it is inappropriate to consider small vessels as the only nutritionally useful component of the vasculature. In the sarcoma the fraction of viable tissue occupied by the capillary space was between 1 and 2%, with no obvious size dependence.

In conclusion, the fluorescent dye H33342 allows a more detailed study of the vascular patterns in tumours than is possible with isotopic methods. Although it may have a slight effect on vessel tone, it gives estimates of the vascular volume perfused at 1 min that are closely similar to those obtained from a 30 min perfusion of labelled red blood cells. It is shown to be a stable cell marker which has a rapid clearance from the blood stream, and hence may be suitable for studies in which multiple injections are used to measure changes in vascular space as time is allowed for opening and closing of vessels.

We are grateful to Dr G. Wilson and Miss A. Lewis for the cytofluorometric measurements. We would also like to thank Mr P. Russell and the animal house staff for care of the mice, Mrs Jean Wilson and Mrs Eileen Marriott for secretarial assistance, and Prof J.F. Fowler and Mr V.S. Randhawa for constructive criticisms. This work was entirely financed by the Cancer Research Campaign.

### References

ARNDT-JOVIN, D.J. & JOVIN, T.M. (1977). Analysis and scoring of living cells according to deoxyribonucleic acid content. *J. Histochem. Cytochem.*, **25**, 585.

CHALKLEY, H.W. (1943). Method for the quantitative morphologic analysis of tissues. *J. Natl Cancer Inst.*, **4**, 47.

CHAPLIN, D.J., DURAND, R.E. & OLIVE, P.L. (1985). Cell selection from a murine tumour using the fluorescent probe Hoechst 33342. *Br. J. Cancer*, **51**, 569.

CHAPLIN, D.J., OLIVE, P.L. & DURAND, R.E. (1987). Intermittent blood flow in a murine tumour. Radiobiological effects. *Cancer Res.*, **47**, 597.

DENEKAMP, J. & HOBSON, B. (1982). Endothelial cell proliferation in experimental tumours. *Br. J. Cancer*, **46**, 711.

DURAND, R.E. & OLIVE, P.L. (1982). Cytotoxicity, mutagenicity and DNA damage by Hoechst 33342. *J. Histochem. Cytochem.*, **30**, 111.

FALK, P. (1978). Patterns of vasculature in two pairs of related fibro-sarcomas in the rat and their relation to tumour responses to single large doses of radiation. *Europ. J. Cancer*, **14**, 237.

FOWLER, J.F. (1983). La Ronde – radiation sciences and medical radiology. *Radiother. Oncol.*, **1**, 1.

GRAY, L.H., CONGER, A.D., EBERT, M., HORNSEY, S. & SCOTT, O.C.A. (1953). The concentration of oxygen dissolved in tissues at the time of irradiation as a factor in radiotherapy. *Br. J. Radiol.*, **26**, 638.

HILL, S.A. & DENEKAMP, J. (1979). The response of six mouse tumours to combined heat and X-rays: Implications for therapy. *Br. J. Radiology*, **52**, 209.

HILL, S.A., FOWLER, J.F., MINCHINTON, A.I., STRATFORD, M.R.L. & DENEKAMP, J. (1983). Radiosensitization of a mouse tumour by Ro-8799 acute and protracted administration. *Int. J. Radiat. Biol.*, **44**, 143.

JIRTLE, R., CLIFTON, K.H. & RANKIN, J.H.G. (1978). Measurement of mammary tumor blood flow in unanaesthetized rats. *J. Natl Cancer Inst.*, **60**, 881.

MURRAY, J.C., RANDHAWA, V.S. & DENEKAMP, J. (1987). The effects of melphalan and misonidazole on the vasculature of a murine sarcoma. *Br. J. Cancer*, **55**, 233.

OLIVE, P.L., CHAPLIN, D.J. & DURAND, R.E. (1985). Pharmacokinetics, binding and distribution of Hoechst 33342. *Br. J. Cancer*, **52**, 739.

PETERSON, H-I. (1978). *Tumour blood circulation*. CRC Press.

PREISLER, H.D. (1978). Alteration of binding of a supravital dye Hoechst 33342 to human leukemic cells by Adriamycin. *Cancer Treatment Rep.*, **62**, 1393.

REINHOLD, H.S. & VISSER, J.W.M. (1983). *In-vivo* fluorescence of endothelial cell muclei stained with the dye Bis-benzamide H33342. *Int. J. Microcirc. Clin. Exp.*, **2**, 143.

RUBIN, R. & CASARETT, G. (1966). Microcirculation of tumours. I Anatomy, function and necrosis. *Clin. Radiol.*, **17**, 220.

SAPIRSTEIN, L.A. (1958). Regional blood flow by fractional distribution of indicators. *Am. J. Physiol.*, **193**, 161.

SIEMANN, D.W. (1982). Potentiation of chemotherapy by hypoxic cell radiation sensitizers – a review. *Int. J. Radiat. Oncol. Biol. Phys.* **8**, 1029.

SOLESVIK, O.V., ROFSTAD, E.K. & BRUSTAD, T. (1985). Vascular structure of the $C_3H$ mammary carcinoma, the B16 melanoma and the Lewis Lung carcinoma in syngeneic, conventional mice and congenitally athymic mice. *Eur. J. Cancer Clin. Oncol.*, **21**, 499.

SOLESVIK, O.V., ROFSTAD, E.K. & BRUSTAD, T. (1982). Vascular structure of five human malignant melanomas grown in athymic nude mice. *Br. J. Cancer*, **46**, 557.

SONG, C.W. & LEVITT, S.H. (1970). Effect of X-irradiation on vascularity of normal tissues and experimental tumour. *Radiology*, **94**, 445.

SONG, C.W. & LEVITT, S.H. (1971). Quantitative study of vascularity in Walker Carcinoma 256. *Cancer Res.*, **31**, 587.

TANNOCK, I.F. (1968). The relation between cell proliferation and the vascular system in a transplanted mouse mammary tumour. *Br. J. Cancer*, **22**, 258.

TANNOCK, I.F. & STEEL, G.G. (1969). Quantitative techniques for study of the anatomy and function of small blood vessels in tumours. *J. Natl Cancer Inst.*, **42**, 771–782.

THOMLINSON, R.H. & GRAY, L.H. (1955). The histological structure of some human lung cancers and the possible implications for radiotherapy. *Br. J. Cancer*, **9**, 539.

THOMLINSON, R.H. & CRADDOCK, E.A. (1967). The gross response of an experimental tumour to single doses of X-ray. *Br. J. Cancer*, **21**, 108.

VAUPEL, P.W., FRINAK, S. & BICHER, H.I. (1981). Heterogeneous oxygen partial pressure and pH distribution in $C_3H$ mouse mammary adenocarcinoma. *Cancer Res.*, **41**, 2008.